# Tenofovir versus entecavir on decreasing risk of HBV-related hepatocellular carcinoma recurrence after liver transplantation

**DOI:** 10.1186/s13027-022-00478-4

**Published:** 2023-01-17

**Authors:** Jianming Yang, Yewu Chen, Haobin Sun, Xijian Zhang, Jianfeng Wang, Zhixing Liang, Binsheng Fu, Tong Zhang, Shuhong Yi, Yinan Deng, Yang Yang

**Affiliations:** 1 Guangdong Provincial Key Laboratory of Liver Disease Research, Department of Hepatic Surgery and Liver Transplantation Center, The Third Affiliated Hospital of Sun Yat-sen University, Guangzhou, China; 2 Guangdong Provincial Key Laboratory of Liver Disease Research, Guangzhou, China

**Keywords:** Tenofovir disoproxil fumarate, Entecavir, Nucleos(t)ide analogues, Hepatocellular carcinoma, Recurrence, Liver transplantation

## Abstract

**Background:**

Recent studies have proved that tenofovir disoproxil fumarate (TDF) is associated with a lower risk of hepatocellular carcinoma (HCC) occurrence in chronic hepatitis B (CHB) patients and HCC recurrence in patients who underwent hepatectomy when compared to ETV. However, it is unclear whether TDF and ETV treatment, which are both recommended as first-line antiviral agents to prevent the hepatitis B (HBV) recurrence after liver transplantation (LT), are associated with equivalent prognosis. We aim to compare risk of HCC recurrence and survival of patients recieving TDF or ETV after LT for HBV-related HCC.

**Method:**

We performed a retrospective study including 316 patients who received treatment with ETV or TDF after LT for HBV-related HCC from 2015 January to 2021 Augest. The Recurrence-free survival (RFS) and overall survival (OS) of TDF and ETV groups were analyzed and compared by propensity score-matched (PSM), multivariable Cox regression analysis, competing risk analysis, sensitivity analyses and subgroup analyses.

**Result:**

Compared with ETV, TDF therapy was associated with significantly higher RFS rates in the entire cohort (*P* < 0.01), PSM cohort (*P* < 0.01) and beyond-Milan cohort (*P* < 0.01). By multivariable analysis, TDF group was associated with significantly lower rates of HCC recurrence (HR, 0.33; 95%CI, 0.14–0.75; *P* < 0.01). In subgroup analyses, the similar results were observed in patients with following tumor characteristics: Maximum diameter plus number of viable tumor ≥ 5, with MIV or MAT, AFP at LT ≥ 20 ng/ml, and well or moderate tumor grade.

**Conclusion:**

Tenofovir decrease risk of HBV-Related Hepatocellular Carcinoma recurrence after liver transplantation compared to Entecavir.

## Introduction

Liver transplantation (LT) is regarded as the radical treatments for hepatocellular carcinoma (HCC), one of the most common causes of cancer-related mortality in the world [1]. However, with the extented indications of LT for HCC, the rate of HCC recurrence after LT is inevitably increasing. It’s reported that the rate of HCC recurrence had reached 20–57.8% in 5 years after transplantation, and the median survival of liver recipients was only 10.6–12.2 months [2–4]. HCC recurrence and metastasis after LT have became the leading factors reducing the curative efficacy of LT.

Chronic hepatitis B (CHB) is one of the leading causes of HCC occurrence world widely, especially in China, about 100 million people are infected with HBV and 20% of them will progress to chronic infection [5]. Evidences showed that high pre-LT HBV DNA levels (> 5log10 copies/ml) and post-LT HBV recurrence are reported as predictors of HCC recurrence after LT [6]. On the contrary, long-term therapy of nucleos(t)ide analogues (NAs) would decreasing the risk of HCC recurrence after LT through reducing HBV load and preventing HBV recurrence [7, 8]. Tenofovir disoproxil fumarate (TDF) and Entecavir (ETV) are both the first-line treatment for the prevention of HBV recurrence after LT in clinical practice guideline because of their equally high antiviral efficacy and high genetic barriers to resistance [9, 10]. Several studys have proved that TDF is associated with a lower risk of HCC occurrence in CHB patients and HCC recurrence in patients who underwent hepatectomy when compared to ETV [11–14]. Nevertheless, it remains unclear whether TDF and ETV treatment are associated with equivalent prognosis in patients who underwent LT for HBV-related HCC.

Thus, we conduct this retrospective cohort study to review and compare the risk of HCC recurrence and survival of patients receiving TDF or ETV therapy after LT for HBV-related HCC.

## Methods

### Study population

We collated 377 consecutive patients received LT for HCC from 2015 January to 2021 Augest at The Third Affiliated Hospital of Sun-Yet Sen University (Guangzhou, China). All the clinical data were obtained from hospital electronic database. Patients with non-HBV-related HCC (n = 30), co-infection of hepatitis C or E virus (n = 5), follow-up time that less than 2 months without any events (recurrence or death, n = 9), regimen which combined with other anti-HBV drugs or did not include ETV and TDF (n = 9), died in 1-month perioperative period (n = 8) were excluded. The final population of 316 patients (44 with TDF, 272 with ETV) were analyzed. This study was approved by the institutional review boards of The Third Affiliated Hospital of Sun-Yet Sen University, Guangzhou, China (Ethics Approval No. [2022]02-028-01).

### Outcomes and follow-up assessment

The primary outcome is HCC recurrence which was defined using the same criteria as for the diagnosis of HCC with Contrast-enhanced computed tomography (CT), ultrasonography or enhanced magnetic resonance imaging (MRI). The index date was defined as the date of LT for HCC. All patient’s recurrence-free survival (RFS) and overall survival (OS) were computed from the index date to the confirmation of recurrence, all-cause mortality, date of secondary-LT for non-HCC, date of antiviral therapy changing or last follow up (2021 October). MELD scoring was applied for assessment of prognosis before LT [15].

All patients have received lowdose hepatitis B immune globulin (HBIG) combining with long-term NAs treatment (TDF, 0.3 g/d; ETV, 0.5 g/d) after LT. According to the Chinese Technical Specifications For Follow-up after Liver Transplantation [16], the follow-up protocol was identical between patients receiving ETV or TDF as follow: Each patients were suggested to follow up once a week within first 3 months after LT, once 2 weeks within forth to sixth months, once a months within seventh to twelfth months and then every 3–6 months. Evaluation item included ultrasonography, Contrast-enhanced CT, liver function tests, serum alpha-fetoprotein (AFP) and blood concentration of Tacrolimus (FK506) or Ciclosporin. Enhanced MRI or Positron emission tomography-computed tomography (PET-CT) was conducted when progression of HCC was not confirmatory by CT during follow-up.

### Statistical analysis

Continuous data were summarized as median (range) without normal distribution, and Student t test and Wilcoxon rank test were applied for comparison of continuous variables with or without normal distribution. Categorical data which were expressed as exact number and proportion were compared using Chi-square and Fisher’s test. Survival curves of RFS and OS were estimated by Kaplan–Meier method and compared by a log-rank test. Multivariable analysis were conducted by Cox regression models, and variables with *P* < 0.05 in Univariables analysis were eligible for the Cox regression models, and the independent risk factors in multivariate analysis were futher used to conduct subgroup analyses. Competing risk analysis was applied to adjust competing risk of the probability of death and secondary LT.

To avoid selection bias and potential confounding, we performed Propensity Score Matched (PSM) analysis. A 1:2 nearest neighbor matching scheme with a caliper size of 0.1 was used to identified the final PSM cohort, propensity scores were computed through the following 21 variables: age; gender; locoregional therapy (LRT) before LT; Maximum tumor diameter; number of lesion; macrovascular invasion (MAV); macrovascular tumor thrombus; microvascular tumor invasion (MIV); satellite nodule; differentiation of tumor; serum levels of HBVDNA, AFP, PT, FIB, INR, ALB, Cr, TB before LT; valley concentration of FK506 or Ciclosporin in 1 month; steatosis of donor liver; cold ischemia time and MELD score. Furthermore, Milan Criteria (MC) was used to stratify the population into two cohort, meeting-MC cohort was considered to be with low risk of HCC recurrence, and beyond-MC cohort was considered to be with high risk of HCC recurrence relatively.

Missing values accounted for 0.1% of the baseline data, which were regarded as random occurrences, and Multivariate Imputation via Chained Equation (MICE) was applied for estimating the missing values. *P* < 0.05 was considered as statistically significant. All statistical analyses were performed using SPSS 25.0 software (SPSS, Inc. an IBM Company, Chicago, IL, USA)and R statistical software, version 4.1.0, (R foundation Inc; http://cran.r-project.org/). The R packages including mice, MatchIt, cmprisk, survival, survminer, tableone, and ggplot2 were used to analyze the statistics and create the figures and tables.

## Results

### Baseline characteristics

The baseline characteristics were presented in Table 1. Among the entire cohort of 306, the median age was 52 years old, ranging from 22 to 77. The median MELD score was 12, ranging from 6.4 to 49.0 points. Because of long-term intake of antiviral drugs before LT, most of patients had relatively low HBVDNA load of ≤ 10^5^ copies/ml (n = 279, 88.2%). Most of tumors have microvascular invasion (n = 196, 62.0%) and nearly half of patients received LRT before LT (n = 149, 47.2%), which include TACE, RFA and gamma knife. Owing to LRT, 8 patients were observed with no viable tumor through CT or MRI imaging and 25 patients were histologically comfirmed to be with no survival tumor. The median maximum diameter of tumor was 3.8 cm, ranging from 0 to 17.4 cm. The average level of serum AFP before sugery was 6551.75 ng/L, ranging from 0.2 to 484, 000 ng/L. For pathology features, the differentiation of tumor were predominantly moderate (n = 223, 70.5%). The average cold ischemia time (CIT) was 374 min, ranging from 215 to 660 min.

**Table 1:** Baseline characteristics of patients recieving TDF or ETV therapy after LT for HBV-related HCC in the entire cohort and PSM cohort

Characteristics	Entire cohort (n = 316)			Propensity socre matched cohort (n = 106)		
	ETV (n = 272)	TDF (n = 44)	*P* value	ETV (n = 68)	TDF (n = 38)	*P* value
*Demographic characteristics*						
Male gender, n (%)	254 (93.4)	41 (93.2)	1.000	63 (92.6)	35 (92.1)	1.000
Age (median [IQR])	52 [46, 59]	50 [44, 54]	0.055	52 [46, 56]	50 [44, 55]	0.410
LRT before LT, n (%)	127 (46.7)	22 (50)	0.806	31 (45.6)	20 (52.6)	0.622
*Tumor characteristics*						
Maximum tumor size (median (IQR)), cm	3.8 (2.1, 6.0)	3.5 (2.5, 5.1)	0.449	35.0 [17.8, 56.2]	34.5 [25.0, 50.0]	0.739
Number of lesion ≥ 3, n (%)	139 (51.1)	21 (47.1)	0.800	32 (47.1)	19 (50.0)	0.930
MAV, n (%)	90 (33.1)	10 (22.7)	0.232	16 (23.5)	10 (26.3)	0.933
MIV, n (%)	179 (65.8)	17 (38.6)	0.001	34 (50.0)	15 (39.5)	0.401
Macrovascular tumor thrombus, n (%)	80 (29.4)	7 (13.6)	0.046	14 (20.6)	6 (15.8)	0.729
Differentiation of tumor, n (%)			0.791			0.639
None survival tumor	23 (8.5)	2 (4.5)		5 (7.4)	2 (5.3)	
Well	22 (8.1)	3 (6.8)		4 (5.9)	3 (7.9)	
Moderate	191 (70.2)	32 (72.7)		46 (67.6)	29 (76.3)	
Poor	36 (13.2)	7 (15.9)		13 (19.1)	4 (10.5)	
Satellite nodule, n (%)	62 (22.8)	7 (15.9)	0.407	13 (19.1)	7 (18.4)	1.000
Beyond MC, n (%)	83 (30.5)	14 (31.8)	1.000	25 (36.8)	11 (28.9)	0.548
*Laboratory findings before LT*						
AFP (median [IQR]), ng/ml	32.8 [5.9, 617.7]	36.1 [6.5, 270.8]	0.820	32.4 [6.2, 544.5]	22.8 [7.4, 183.2]	0.849
PT (median [IQR]), sec	14.9 [13.8, 17.2]	14.8 [13.8, 19.0]	0.602	15.4 [13.7, 17.9]	15.3 [13.9, 19.6]	0.843
FIB (median [IQR]), g/L	2.7 [2.0, 3.7]	2.8 [1.7, 3.7]	0.805	2.5 [1.9, 3.8]	2.7 [1.7, 3.5]	0.963
INR (median [IQR])	1.2 [1.0, 1.4]	1.2 [1.1, 1.7]	0.353	1.2 [1.0, 1.5]	1.2 [1.1, 1.6]	0.624
TB (median [IQR]), umol/L	24.0 [13.3, 42.2]	23.1 [12.4, 84.4]	0.787	21.4 [13.5, 49.0]	21.7 [12.8, 63.0]	0.715
ALB (median [IQR]), g/L	36.3 [32.3, 40.4]	34.9 [31.4, 38.7]	0.179	35.6 [31.6, 40.5]	35.9 [32.8, 39.5]	0.911
Cr (median [IQR]), umol/L	73.0 [63.0, 84.0]	70.5 [60.0, 88.8]	0.953	77.0 [65.0, 84.2]	70.5 [60.5, 85.8]	0.420
MELD score (median [IQR])	9.9 [7.6, 14.1]	11.0 [7.5, 20.5]	0.216	11.6 [11.6, 43.6]	11.6 [11.6, 43.6]	0.417
HBVDNA ≥ 5log10 copy/ml, n (%)	32 (11.8)	5 (11.4)	1.000	8 (11.8)	5 (13.2)	1.000
HBV recurrence after LT	55 (20.2)	3 (6.8)	0.055	10 (14.7)	3 (7.9)	0.474
*Other characteristics*						
Steatosis of donor liver (median [IQR]),%	0.0 [0.0, 9.0]	4.0 [0.0, 19.2]	0.015	4.0 [0.0, 10.0]	4.5 [0.0, 16.8]	0.630
Valley concentration of FK506 (> 10 ng/ml)or Ciclosporinin (> 300 ng/ml)in 1 month, n (%)	64 (23.5)	6 (13.6)	0.204	9 (13.2)	6 (15.8)	0.943
CIT (median [IQR])	360 [340,4 17]	359.0 [300, 391]	0.110	364 [323, 412]	355.0 [300, 388]	0.141
Follow up time (median[IQR]), month	24.0 [13.0, 39.0]	15.5 [8.0, 29.0]	0.004	26.0 [16.8, 39.8]	16.0 [9.2, 29.0]	0.010

*LRT*, locoregional treatment; *LT*, liver transplantation; *MAV*, macrovascular invasion; *MIV*, microvascular invasion; *MC*, Milan criteria; *AFP*, alpha-fetoprotein; *PT*, prothrombin time; *FIB*, fibrinogen; *INR*, international normalized ratio; *TB*, total bilirubin; *ALB*, albumin; *Cr*, creatinine; *MELD*, Model for End-stage Liver Disease; *CIT*, cold ischemia time; *IQR*, interquartile ranges

Patients were divided into groups regarding their antiviral therapy after LT. The median ages of ETV and TDF groups were 52 and 50 years, respectively. Compared to ETV group, TDF group had significantly less patients with MIV (65.8% vs 38.6%, *P* < 0.05) and macrovascular tumor thrombus (29.4% vs 13.6%, *P* < 0.05), and significantly more severe steatosis of donor liver (0% vs 4%; *P* < 0.05), and shorter follow up time (15.5 months vs 24.0 months), which were mostly balanced in the PSM cohort. The other variables have no significant difference between two groups.

### Survival analysis of the entire cohort

**Fig. 1:**
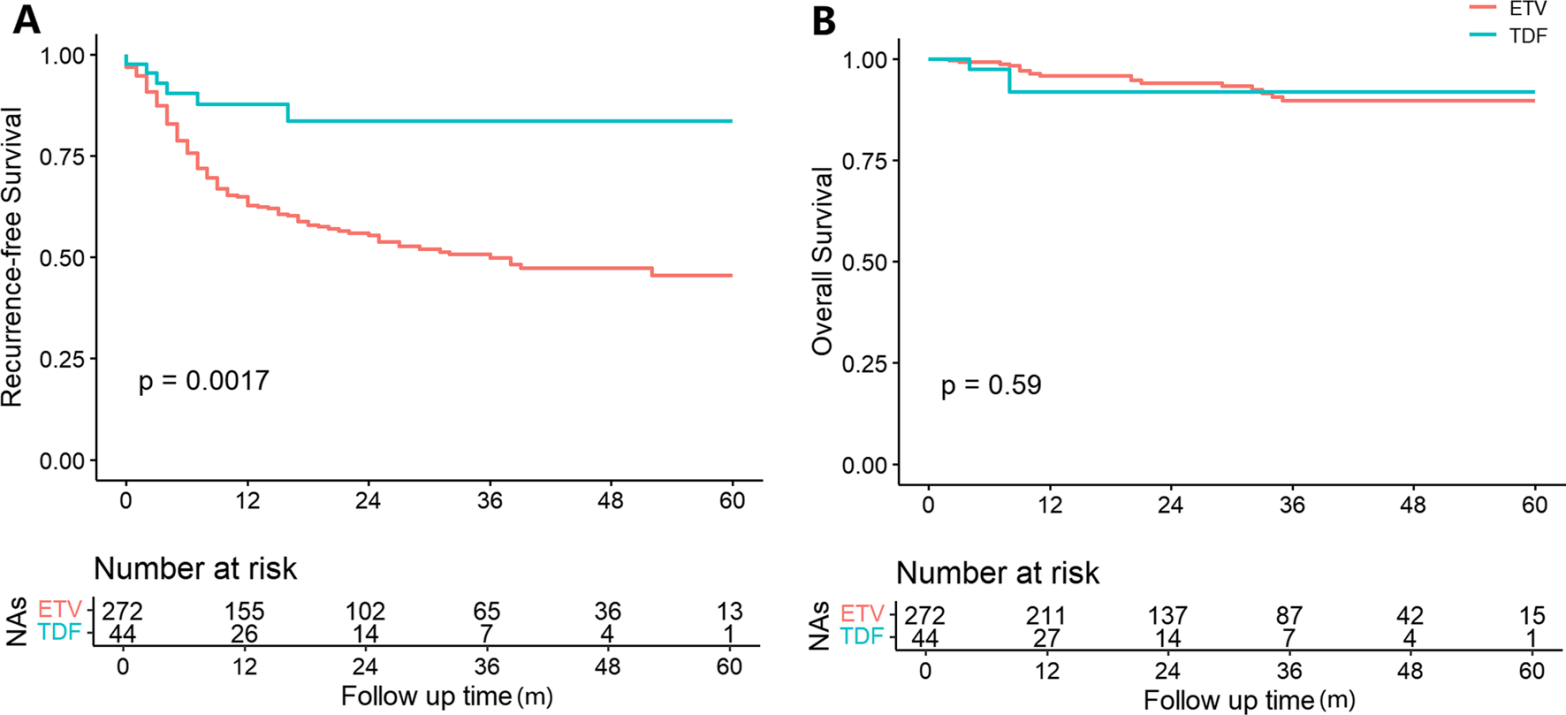
Recurrence-free and overall survival of the entire cohort of patients receiving TDF or ETV therapy after LT for HBV-related HCC. TDF = tenofovir disoproxil fumarate; ETV = entecavir; LT = liver transplantation; HBV = hepatitis B virus; HCC = hepatocellular carcinoma. **A**. Comparison of Recurrence-free Survival in entire cohort. **B**. Comparison of Overall Survival in entire cohort

In the entire cohort, 133(42.1%) patients developed HCC recurrence and 21 (6.6%) patients died. The TDF group showed a significantly better RFS compared with the ETV group (83.6% vs. 45.4% at 5-year *P* < 0.01, Fig. 1A). However, OS was not significantly different between two group (*P* = 0.59, Fig. 1B).

In the multivariable Cox regression model, the TDF group was associated with a significantly lower risk of HCC recurrence than the ETV group (hazard ratio [HR], 0.33;95% confidence interval [CI], 0.14–0.75; *P* < 0.01), which was independent to other predictive factors (Table 2). Other significant factors associated with HCC recurrence were maximum diameter plus number of lesion (2.32; 1.32–4.09, *P* < 0.01), MIV (2.55; 1.48–4.39, *P* < 0.01), Macrovascular tumor thrombus (1.84; 1.25–2.71, *P* < 0.01), AFP (2.07; 1.38–3.13, *P* < 0.01), and differentiation of tumor (1.23; 1.06–1.43, *P* < 0.01). Moreover, this result remains stable after adjusting competing risk of secondary-LT and non-HCC-related death (0.32; 0.13–0.79, *P* = 0.014).

**Table 2:** Univariable and Multivariable analysis for hepatocellular carcinoma (HCC) recurrence after LT in the entire cohort

Characteristics	Univariate			Multivariate		
	HR	95%CI	*P*	aHR	95%CI	*P*
Male gender (vs female)	3.95	1.25–12.41	0.019	2.05	0.64–6.58	0.230
Age ≥ 60y (vs < 60y)	0.84	0.55–1.26	0.394			
NAs (TDF vs ETV)	0.29	0.13–0.67	0.003	0.33	0.14–0.75	0.008
LRT before LT	0.90	0.64–1.27	0.558			
Maximum diameter plus number of lesion > 5 (vs ≤ 5)	4.28	2.50–7.34	< 0.001	2.32	1.32–4.09	0.004
MAV	2.27	1.61–3.19	< 0.001	1.22	0.84–1.78	0.302
MIV	5.41	3.25–9.00	< 0.001	2.55	1.48–4.39	< 0.001
Macrovascular tumor thrombus	3.97	2.81–5.59	< 0.001	1.84	1.25–2.71	0.002
AFP > 20 ng/ml (vs ≤ 20 ng/ml)	3.12	2.10–4.63	< 0.001	2.07	1.38–3.13	< 0.001
PT > 14.5 s (vs ≤ 14.5 s)	1.03	0.73–1.45	0.884			
FIB > 1.7 g/L (vs ≤ 1.7 g/L)	1.56	0.95–2.56	0.08			
INR > 1.1 (vs ≤ 1.1)	0.92	0.65–1.31	0.662			
TB > 34.2umol/L (vs ≤ 34.2umol/L)	1.28	0.91–1.80	0.154			
ALB > 36 g/L (vs ≤ 36 g/L)	0.79	0.56–1.11	0.181			
Cr > 116umol/L (vs ≤ 116umol/L)	0.90	0.42–1.93	0.788			
MELD > 15 (vs ≤ 15)	1.14	0.77–1.67	0.515			
HBVDNA > 5log10 copies/ml (vs ≤ 5log10 copies/ml)	1.14	0.68–1.89	0.626			
Poor differentiation	1.44	1.26–1.65	< 0.001	1.23	1.06–1.43	0.005
Satellite nodule	2.21	1.54–3.18	< 0.001	1.17	0.80–1.71	0.418
Valley concentration of FK506 (> 10 ng/ml)or Ciclosporinin (> 300 ng/ml) in 1 month after LT	1.25	0.85–1.84	0.255			
Steatosis of donor liver	0.79	0.56–1.11	0.172			
CIT > 300 min (vs ≤ 300 min)	1.32	0.65–2.70	0.446			

*LRT*, locoregional treatment; *LT*, liver transplantation; *MAV*, macrovascular invasion; *MIV*, microvascular invasion; *MC*, Milan criteria; *AFP*, alpha-fetoprotein; *PT*, prothrombin time; *FIB*, fibrinogen; *INR*, international normalized ratio; *TB*, total bilirubin; *ALB*, albumin; *Cr*, creatinine; *MELD*, Model for End-stage Liver Disease; *CIT*, cold ischemia time; *HR*, hazard ratio; *CI*, confidence interval; *aHR*, adjusted hazard ratio

### Survival analyses of PSM cohort and meeting or beyond-MC cohort

**Fig. 2:**
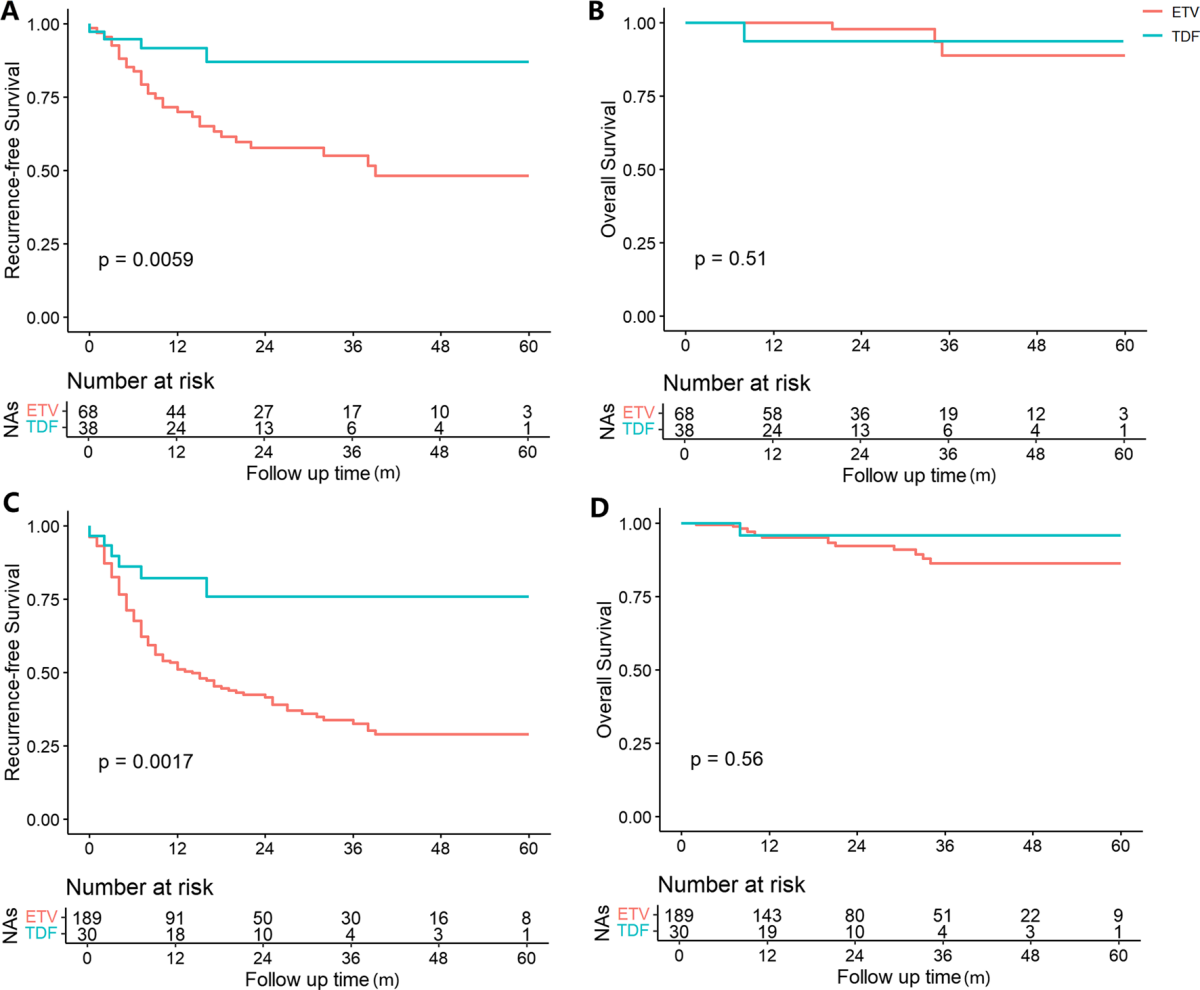
Recurrence-free and overall survival of the PSM and beyond-MC cohort of patients receiving TDF or ETV therapy after LT for HBV-related HCC. PSM = propensity score matching; MC = Milan criteria. **A**. Comparison of Recurrence-free Survival in PSM cohort. **B**. Comparison of Overall Survival in PSM cohort. **C**. Comparison of Recurrence-free Survival in beyond-MC cohort. **D**. Comparison of Overall Survival in beyond-MC cohort

Among the PSM cohort (38 with TDF vs 68 with ETV), the TDF group had significantly better RFS rate than the ETV group (0.26; 0.09–0.73; *P* < 0.01; Fig. 2A). The estimated 5-year RFS rates of TDF and ETV group were 87% versus 48%. In the meeting-MC cohort, the RFS curve showed no significant difference between TDF and ETV group (*P* = 0.25). In contrast, the TDF group presented significantly better RFS rate than the ETV group in the beyond-MC cohort (0.29; 0.13–0.66, *P* < 0.01; Fig. 2C). In order to further reduce the effect of confounding, we conducted a PSM analysis of beyond-MC cohort, which showed similar result to that of before-PSM cohort (*P* = 0.046). Nevertheless, the OS analyses again shown no significant difference between two drugs in the PSM cohort (*P* = 0.51. Figure 2B) and beyond-MC cohort (*P* = 0.56; Fig. 2D).

### Subgroup and sensitivity analyses

**Fig. 3:**
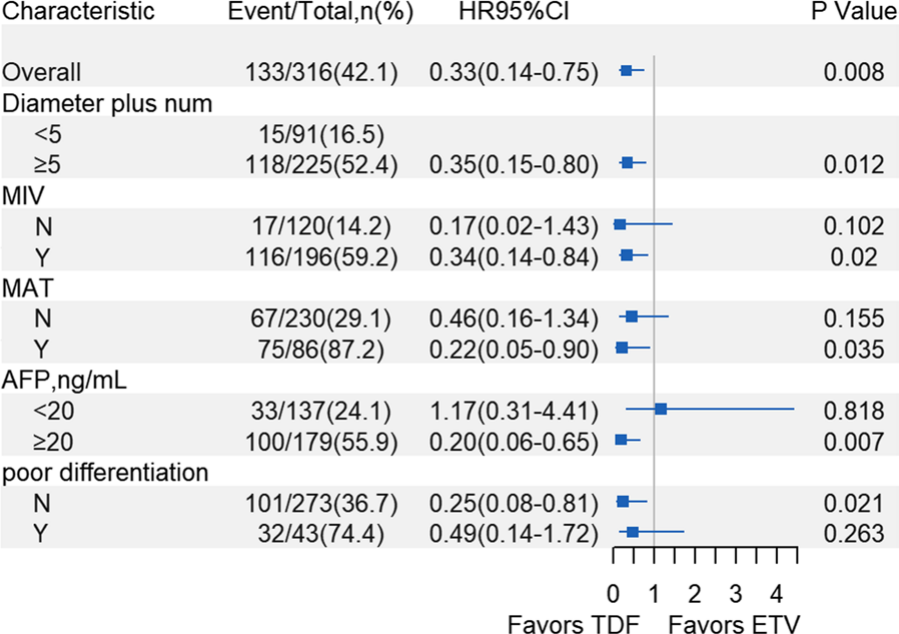
Forestplot for subgroup analysis of the comparison between TDF and ETV. HR, hazard ratio; CI = confidence interval; Diameter plus num = maximum size plus number of viable tumor; MIV = microvascular invasion; MAT = macrovascular tumor thrombus; AFP = alpha- fetoprotein

As presented in subgroup analyses (Fig. 3), TDF therapy showed a significantly better effect on reducing risk of HCC recurrence in patients with following tumor characteristics: Maximum diameter plus number of viable tumor ≥ 5, with MIV or MAT, AFP at LT ≥ 20 ng/ml, and well or moderate tumor grade.

In order to decrease potential confounding, we conducted the following sensitivity analyses:first, given that TDF was applied later than ETV in China, we excluded cases before the first use of TDF (ie, cases before 2016, n = 22), and our results remianed unchanged (aHR, 0.34; 95%CI, 0.15–0.77, *P* = 0.010). What’s more, when excluded those with a follow-up time less than 1 year (n = 78), the TDF again associated with a lower risk of HCC recurrence compared with ETV (aHR, 0.15; 95%CI, 0.04–0.62, *P* = 0.009), and the difference of follow-up times was eradicated (28 month of TDF vs 32 month of ETV, *P* = 0.092, not shown in data). Second, we excluded those who have had received LRT ≥ 3 times (n = 48) and observed that the result were similar (aHR, 0.38; 95%CI, 0.15–0.94, *P* = 0.037); Finally, our result were again similar when excluded cases that experienced intrahepatic recurrence (n = 87, aHR, 0.13; 95%CI, 0.02–0.96, *P* = 0.046). When excluded cases of extrahepatic recurrence (n = 100), the anti-tumor effect of TDF was not observed (aHR, 0.30; 95%CI, 0.07–1.28, *P* = 0.102).

### HBV recurrence after LT

HBV recurrence was defined as reapperance of HBsAg, HBeAg or HBV-DNA during follow up time. In our study, 58 patients were observed to develop HBV recurrence and there was a trend that TDF group experienced less HBV recurrence compared with ETV (6.8% vs 20.2%, *P* = 0.055; Table 1).

## Discussion

Recent studies has shown that TDF treatment was associated with lower risk of HCC than ETV in patients with chronic HBV Infection, and decrease recurrence of HBV-related HCC after hepatectomy [11–14, 17–22]. However, there is no article reporting the same effect of TDF on those patients who underwent LT as far. The pathophysiological condition of these patients are completely different from that of patients with mere cirrhosis or underwent hepatectomy. Even though LT is supposed to completely remove the tumors, there is still opportunity for HCC to progress when considering the recurrence of HBV, circulatory tumor cells and change of immune-microenvironment. Theoretically, in liver transplantation, liver as the primary reservoir of HBV is completely removed, and serum HBsAg or HBV-DNA could not be detected in most LT-receivers. However, HBV from extrahepatic reservoirs still remains as the source of HBV recurrence, which might cause HCC recurrence after LT [6]. Therefore, in order to curb HBV and HCC recurrence after LT, it is necessary to explore which antiviral drug is more efficient.

In our cohort study, we analyzed patients who were treated with TDF (n = 44) and ETV (n = 276) after LT for HBV-related HCC and found that TDF therapy was associated with a significantly lower risk of HCC recurrence than ETV treatment, which was observed in entire cohort, PSM cohort, beyond-MC cohort, multivariate analysis, competing risk analysis, subgroup analyses and sensitivity analyses. Other independent risk factors of HCC recurrence, such as microvascular invasion, were consistent with present studies [23]. In subgroup analyses (as Fig. 3 shows), the anti-tumor effect of TDF was mostly observed in patients with more-aggressive HCC. For these patients, We speculate that the circular HBV-DNA had integrated into the extrahepatic tumor cells [24], and increasing viral load caused by HBV recurrence might contribute to the extrahepatic HCC recurrence [25]. TDF may perform a more effective inhibition on HBV replication and recurrence than ETV. The result of sensitivity analyses, which excluded cases of intrahepatic or extrahepatic HCC recurrence, also support this speculation.

The results of the tumor differentiation subgroup were unexpected, which could be ascribed to the fact that preoperative LRT might cause poor-differentiated tumors to be completely necrotic and fall into well-or-moderate-differentiated group. Indeed, the recurrence rate of well-or-moderate-differentiated group have reached up to 36.7% (Fig. 3), which indicated that this factor might not be able to predict low HCC recurrence risk or well prognosis in our study. In order to reduce confounding from LRT, we performed a sensivity analysis that excluded any patients who received LRT ≥ 3 times (n = 48) and observed that the result were similar (aHR, 0.38; 95%CI, 0.15–0.94, *P* = 0.037).

Another negative result of overall survival analyses could be attributed to the small sample size and short follow-up duration, therefore larger scale and longer follow-up duration are mandatory. Moreover, owing to salvage treatments including locoregional and systemic therapy after HCC recurrence, the OS rate of TDF and ETV groups might incline to be consistent, which could also partly explain the negative result of OS analyses.

It is noteworthy that the follow-up times of TDF group was shorter than ETV group, which could be attribute to the later application of TDF in China. Therefore, our results should be interpreted with caution in that some patients in TDF group are supposed to experience HCC recurrence following prolonged follow-up. In order to reduce bias from different follow-up, we conducted a sensitivity analysis that excluded cases before the first use of TDF and those with a follow-up times less than 1 year, and the TDF still associated with a lower risk of HCC recurrence compared with ETV.

According to the present studies, two potential mechanisms may explain our result. Firstly, post-LT HBV recurrence is known as a risk factor of HCC recurrence after LT [6], and TDF might reduce HCC recurrence via inhibiting HBV recurrence. TDF and ETV are not completely similar types of NAs in that TDF belongs to nucleotide analogues and ETV belongs to nucleoside analogues. Nucleotide analogues could cause higher serum interferon-λ3 level, which was proved to have a strong effect on tumor or HBsAg inhibition, and immune modulation [26–28]. In our study, TDF showed a better trend of effect on inhibiting HBV recurrence than ETV, which was comparable with present study [29] and supports these postulation. Secondly, as Murata K’s study showed [30], interleukin (IL)-10 inhibit antigen-specific CD8+ −T cells and IL-12 directly stimulates T cells and NK cells to induce IFN-γ.Pretreatment of peripheral blood mononuclear cells with TDF inhibited production of IL-10, but induced production of IL-12p70 and tumor necrosis factor (TNF)-αin a dose-dependent manner, which was not observed with ETV.

Nevertheless, there are limitations in our study. The major one is that as a retrospective study with small sample size based on observational data, our study may be subjected to selection bias and confounding, though we applied various statistical approaches including multivariable adjustment, PSM, competing risk analysis, subgroup analyses and sensitivity analyses to adjust the differences of clinical characteristics between ETV and TDF group, unmeasured biases and confounding still exist. In addition, some patients get the antiviral drugs from other hospital or pharmacy after discharging, which led to a consequence that we failed to monitor the long-term antiviral regimen of these patients after operation, thus the adherence and duration time of antiviral therapies couldn’t be guaranteed. Poor adherence to antiviral therapies without our monitoring may cause different timing of two drugs and increase risk of HBV-relapse-induced HCC recurrence eading to bias of our results.

## Conclusion

In summary, TDF decrease risk of HBV-Related Hepatocellular Carcinoma recurrence after liver transplantation compared to ETV. Our findings may be of great significance in guiding the use of antiviral regimen after liver transplantation.

## Data Availability

The data that generate the results of this study are available on request from the corresponding author: Yang yang. Department of Hepatic Surgery and Liver Transplantation Center, The Third Affiliated Hospital of Sun Yat-sen University, Guangzhou, China.
